# Effectiveness of Breast Density Educational Interventions on Mammography Screening Adherence Among Underserved Latinas: A Randomized Controlled Trial

**DOI:** 10.1089/jwh.2024.0273

**Published:** 2025-04-02

**Authors:** Jessica D. Austin, Sarah M. Jenkins, Vera J. Suman, Jennifer L. Ridgeway, Bhavika K. Patel, Karthik Ghosh, Deborah J. Rhodes, Bijan J. Borah, Aaron D. Norman, Edna P. Ramos, Matt Jewett, Crystal R. Gonzalez, Valentina Hernandez, Davinder Singh, Carmen Radecki Breitkopf, Celine M. Vachon

**Affiliations:** ^1^ Division of Epidemiology, Quantitative Health Sciences, Mayo Clinic, Arizona, USA.; ^2^ Division of Clinical Trials and Biostatistics, Quantitative Health Sciences, Mayo Clinic, Rochester, Minnesota, USA.; ^3^ Robert D. and Patricia E. Kern Center for the Science of Health Care Delivery, Mayo Clinic, Rochester, Minnesota, USA.; ^4^ Department of Radiology, Mayo Clinic, Phoenix, Arizona, USA.; ^5^ Department of Medicine, Mayo Clinic, Rochester, Minnesota, USA.; ^6^ Yale College of Medicine, New Haven, Connecticut, USA.; ^7^ Department of Surgery, Mayo Clinic, Phoenix, Arizona, USA.; ^8^ Department of Administration, Mountain Park Health Center, Phoenix, Arizona, USA.; ^9^ Department of Integrated Nutrition Services and Collaborative Research, Mountain Park Health Center, Phoenix, Arizona, USA.; ^10^ Division of Epidemiology, Quantitative Health Sciences, Mayo Clinic, Rochester, MN, USA.

**Keywords:** Latinas, breast density, educational intervention, mammography adherence

## Abstract

**Background:**

Latinas with mammographic dense breasts are at increased risk of breast cancer. This randomized controlled trial tests the effectiveness of three breast density (BD) educational approaches on adherence to subsequent mammography screening among Latinas receiving care at a Federally Qualified Health Center (FQHC).

**Measure(s):**

Adherence was ascertained using electronic health record and survey responses. Kaplan–Meier estimates of the time to subsequent mammogram were used to obtain adherence rates at 1- and 2-years post baseline mammogram. Cox modeling assessed whether adherence differed by patient characteristics or group assignment.

**Results:**

This analysis was limited to Latinas enrolled between October 27, 2016, and December 21, 2018 (*n* = 946; 66.1% <50 years of age, 53.5% with dense breasts). Adherence rates at 1 year was 24.8% increasing to 51.7% by year 2. Latinas randomized to the Promotora + brochure + letter arm (hazard ratio [HR]: 1.09 [95% confidence interval [95% CI] 0.87 to 1.36]) or brochure + letter (HR: 1.03 [95% CI 0.82 to 1.29]) arm were not more likely to be adherent to subsequent mammography compared to the letter only arm (*p = 0.76)*. Adjusting for age and study group, having more prior mammograms, being “extremely likely” to get an annual mammogram, and having more confidence to get an annual mammogram at baseline were significant drivers of subsequent adherence.

**Conclusion:**

Informational interventions targeting BD education alone are unlikely to significantly improve adherence to subsequent mammography among Latinas receiving care in FQHCs.

## Introduction

Breast cancer (BC) remains a leading cause of death among Latina women in the United States.^1^ Mammographic breast density (MBD) is a measure of fibroglandular tissue composition on a mammogram that can reduce the ability to detect tumors and is one of the strongest independent predictors of BC.^2–4^ Dense breasts are common,^5^ but the distribution of breast density (BD) categories varies across racial and ethnic groups.^6^ For instance, some studies show that Latina populations have higher BD compared to other ethnic groups,^7^ while other studies show that Latina women are less likely to have dense breasts.^8^

State and federal legislation mandate notifying women about their MBD status with the goal of enhancing awareness and knowledge of what MBD is and its association with BC risk.^9–11^ Implementation of these laws vary, with some limiting notification to women with dense breasts only and others notifying all women regardless of their MBD status. A key impetus of these laws is to empower women to become more involved in future screening plans, but there is heightened concern that notifications may lead to undesirable outcomes and exacerbate existing disparities.^12,13^ For instance, the Food and Drug Administration (FDA) recently required specific language including the potential need for additional imaging for those with dense breasts,^10^ despite clear clinical pathways and insufficient evidence supporting the benefits of supplemental imaging based on MBD alone.^12,14^ Supplemental screening is also not covered by insurance or readily available in safety-net settings, such as Federally Qualified Health Centers (FQHCs) who provide critical primary care services to disadvantaged populations.^12,15^ Moreover, it is well known that women understand and react to BD notification differently, which can influence future screening decisions.^15–17^

Latinas experience inequities across the BC care continuum that may be further exacerbated by BD notification legislation.^16–19^ Several studies including Latina women have demonstrated low levels of BD awareness and knowledge,^20–24^ as well as heightened feelings of worry and anxiety,^18,25^ which have been attributed to poor readability and understandability of BD notification.^26,27^ This has led to increasing calls for BD educational efforts to ensure that BD notifications are accessible and understandable to all women in order to lead to desired effects.^16^ It is also well established that Latinas are less likely to be adherent to BC screening and less likely to receive supplemental screening due to various socioeconomic and structural factors, including lack of availability and insurance coverage, transportation barriers, language barriers, and low health literacy.^1,28–36^ To this end, more research is needed among populations most susceptible to BC disparities that address both psychological and behavioral outcomes associated with BD notification.

Few trials aimed at improving BD notification include under- or uninsured Latina women and mainly target improvements in cognitive (i.e., knowledge, awareness, and intention) or psychological outcomes (i.e., worry and anxiety) rather than behavioral outcomes.^15,37^ Interpersonal cancer education interventions delivered by a community health worker or *Promotoras* are increasingly shown to be effective at improving BC screening by addressing barriers including low literacy and knowledge deficits.^38–43^ One-on-one education delivered by a Promotoras may help to address known gaps in BD notification practices among populations most susceptible to disparities in BC screening and outcomes. The purpose of this study was to test the effectiveness of three different BD notification approaches on adherence to attending the next routine screening mammogram or subsequent mammogram. We hypothesized that Latinas randomized to receive a notification letter accompanied by written education materials, and an interaction with a Promotoras would be more likely to attend their next mammography screening appointment compared to Latinas receiving either the notification letter alone or with a written educational brochure.

## Methods

Latinas Learning about Breast Density (LLEAD) is a three-arm randomized controlled trial comparing the effectiveness of three educational approaches on behavioral and psychological outcomes.^19^ This work was supported by the National Institute on Minority Health and Health Disparities (R01MD009682). We report the results of the trial on adherence defined as completion of one’s next routine or subsequent mammogram between 10 months and up to 26 months post-baseline. This range was selected given differing guideline recommendations ranging from annual to biannual mammography screening based on age and medical history.^44–46^ Key outcomes including changes in knowledge, awareness, and anxiety are reported separately.

### Setting

This study was conducted at Mountain Park Health Center’s (MPHC) Baseline Clinic, the largest FQHC in Phoenix, Arizona. MPHC Baseline Clinic provides primary preventive care services, including onsite mammography screening to nearly 2000 women per year (85% Hispanic), regardless of their ability to pay. MPHC guidelines for BC screening recommend initiation of screening for all women 40 years of age or older. In October 2014, Arizona passed BD notification legislation mandating that women with heterogeneously dense or extremely dense breasts (BI-RADS C or D) receive a written letter notifying them of their MBD status, implications of BC risk, and detection. MPHC, however, decided to inform all women regarding the implications of BD *via* mailed written notification letter, regardless of MBD status.

### Recruitment and randomization

**FIG. 1.**
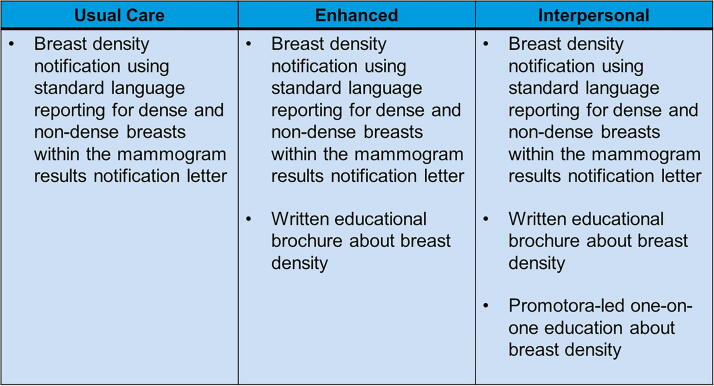
Description of intervention arms.

Eligibility criteria included English- or Spanish-speaking women, aged 40–74 years (per the US Preventive Services Task Force) presenting for a screening (vs. diagnostic) mammogram. While the target population for the study was Latinas, ethnicity was not among the inclusion/exclusion criteria to allow all interested women the opportunity to receive the intervention and its potential benefits. Using clinic schedules, we identified and contacted eligible women by phone at least a week prior to their appointment to ascertain their interest in the study. Those expressing interest were met at their appointment by a bilingual study coordinator to describe the purpose of the study and obtain written informed consent. Those consenting to the study were randomized 1:1:1 to usual care (UC; a standard mailed notification letter), enhanced care (ENH; notification letter + educational brochure), or interpersonal care (INT; notification letter + educational brochure + telephone-based Promotora education). Randomization was performed using a stratified block (size of 6) schema by age (≥50 vs. <50), ethnicity (Latina vs. non-Latina), and language preferences (Spanish vs. English). Figure 1 provides a brief overview of the intervention study arms. All materials were available in both English and Spanish, and additional interventional details are reported previously (Clinicaltrials.gov, R01MD009682).^19^ Study staff were blinded to study group assignment. This study was approved by the Mayo Clinic institutional review board.

### Intervention

#### Usual care

The UC group received the standard clinical BD notification letter from MPHC, which included statements on masking, BC risk, and encouraged women to speak with their provider if they had any questions or concerns.

##### Enhanced

The ENH group received the standard clinical BD notification letter and a study educational brochure developed by the study team alongside patient education experts. The brochure defined MBD and its implications as well as importance of annual screening mammography for early cancer detection.

##### Interpersonal

In addition to the standard clinical notification letter and study educational brochure, Latinas in the INT group received telephone-based education by a Promotora ∼2 weeks following randomization to allow time for the letter and brochure to be received. Informed by the Information-Motivation-Behavioral Skills health behavior model,^47^ the Promotora reviewed information in the letter and brochure, elicited questions, engaged in conversation around breast health and MBD, and delivered motivating messages related to self-care and BC screening.

### Surveys

Surveys were administered at three time points by a bilingual member of the study team: enrollment (T0), 2 weeks to 6 months after intervention delivery (T1), and ∼1-year post randomization (T2). This analysis uses self-reported data from the T0 and T2 survey. The T0 survey included demographic characteristics, family history of BC, mammography screening history (approximate lifetime number of mammograms, number of times recalled, and number of breast biopsies), and psychosocial measures from existing measures when available. Psychosocial measures assessed cancer beliefs (perceived risk,^48^ perceived worry about BC,^49^ confidence to get an annual mammogram (self-efficacy), and likelihood of continuing to get annual mammograms (intention). Perceived risk of BC was measured using both a 0–100% response (percent *lifetime risk*) and a 5-point verbal scale ranging from “very low” to “very high” (*ordinal lifetime risk*).^48^ Perceived worry about BC was assessed by asking how frequently one worries about getting BC someday, from “not at all” to “almost all the time”.^49,50^ Confidence (self-efficacy) to get an annual mammogram was measured on a scale of 1 to 10, with higher scores indicating higher levels of confidence to get an annual mammogram. The 10-point scale was chosen considering language and literacy levels of the participants. Likelihood of continuing annual mammograms (intention) was assessed using a five-point Likert scale ranging from “not at all likely” to “extremely likely”.^50^

The T2 survey asked participants if they had scheduled their follow-up mammogram and if they made the appointment at MPHC or another facility. Those who did not schedule their follow-up appointment were asked to provide an open-ended response for the main reason for not scheduling the appointment. Surveys were available in English and professionally translated into Spanish and included items from previously published measures when available. Participants received $25 for completing each survey. Other key outcomes collected in the T1and T2, including anxiety, knowledge, awareness, and satisfaction, are reported separately.^51^

### Electronic health record and mammography data

Baseline patient characteristics and mammographic findings were abstracted from the MPHC electronic health record (EHR) and PenRad reporting and tracking system. Specifically, insurance status within +/- 90 days of consent was categorized as uninsured (sliding scale, Well Woman Program) and insured. Body mass index was categorized as normal/underweight (<25), overweight (25–29), and obese (30+).^52^ Participants with a BI-RADS category of C/heterogeneously dense or D/extremely dense were classified as having dense breasts, and those with a category A/almost entirely fatty or B/scattered fibro glandular densities were classified as nondense.^53^

### Adherence

Adherence to attending the next routine screening mammogram, or subsequent mammogram, was ascertained using both the EHR and responses to the T2 survey regarding if and when the subsequent mammogram was performed. Since recommended screening intervals differ by major consensus groups, we estimated adherence as a time-to-event outcome and report the estimated cumulative percentage of participants with a completed subsequent screening mammogram at 14- and at 26-months post-baseline mammogram, herein after referred to as 1-year and 2-year adherence, respectively.

### Data analysis

Adherence to attending the next routine or subsequent screening mammogram was the primary outcome for these analyses. Any mammogram performed <10 months post-baseline was not considered a subsequent screening mammogram to avoid capturing diagnostic mammograms. Adherence rates were estimated using the Kaplan–Meier method, censoring participants on March 1, 2020, if they did not have a mammogram on or before that date due to mammography access disruptions caused by the COVID-19 pandemic. To ensure sufficient follow-up for estimating adherence at 1 year, participants with <14 months of follow-up prior to March 1, 2020, were excluded from the analysis (excludes those enrolled in 2019 or later). Cox modeling was performed to assess whether time to subsequent screening mammogram differed with respect to study arm as well as for patient characteristics after adjusting for age and study group. Hazard ratios (HRs) and 95% confidence intervals (CIs) were reported. Given the differential screening recommendations for women under age 50, these analyses were repeated post hoc for the subgroup <50 years of age and the subgroup age 50 or older. Chi-square tests or Wilcoxon rank-sum tests were used to assess difference between those who stated they were “extremely likely” to continue getting an annual mammogram differ from those who reported otherwise. *P* values less than 0.05 were considered statistically significant. All quantitative analyses were performed using SAS version 9.4 (SAS Institute Inc., Cary, NC). Finally, open-ended responses to the item on the T2 survey “What would you say is the main reason you have not made an appointment for a mammogram?” were analyzed using conventional content analysis, among nonadherent participants who self-reported not having had a screening mammogram since enrollment.^54^

## Results

**FIG. 2.**
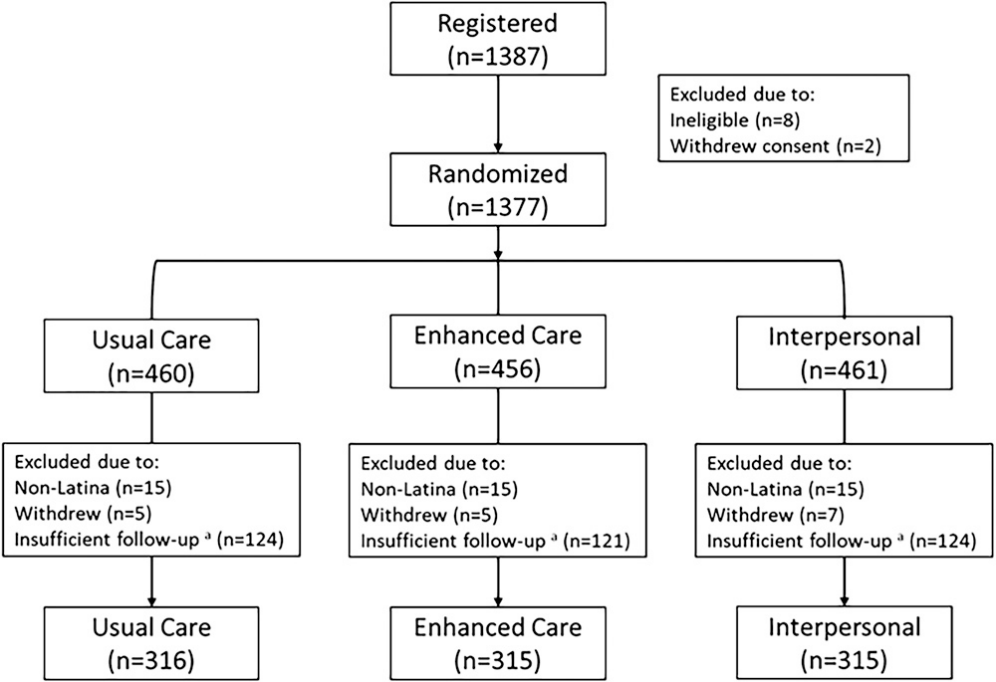
CONSORT diagram.

Between October 2016 and October 2019, 1387 women were enrolled in the trial. This analysis is limited to 946 women randomized prior to 2019, in an attempt to limit the impact of the COVID-19 pandemic on mammography screening adherence as these women had at least 14 months of follow-up available before March 1, 2020, when the pandemic altered routine care practices (Consort Diagram Fig. 2).

As shown in Table 1, 66.1% were under the age of 50, 69.2% had less than a high school education, 19.0% were insured, and 92.7% completed their consent in Spanish. More than half were categorized as having dense breasts (53.5%). Additionally, 82.3% reported having a prior mammogram, 79.9% said they were “extremely likely” to continue getting annual mammograms, 57.3% “rarely” or “not at all” worry about BC, and 76.3% perceived their lifetime chance of developing BC as 10% or less.

**Table 1.** Summary of LLEAD Participant Characteristics at Baseline by Intervention Status

	Usual (*n* = 316)	Enhanced (*n* = 315)	Interpersonal (*n* = 315)	Total (*n* = 946)
Age				
40–44	112 (35.4%)	121 (38.4%)	109 (34.6%)	342 (36.2%)
45–49	98 (31.0%)	87 (27.6%)	98 (31.1%)	283 (29.9%)
50–54	50 (15.8%)	57 (18.1%)	62 (19.7%)	169 (17.9%)
55+	56 (17.7%)	50 (15.9%)	46 (14.6%)	152 (16.1%)
Educational status				
Less than high school	205 (65.5%)	209 (66.6%)	237 (75.5%)	651 (69.2%)
High school or more	108 (34.5%)	105 (33.4%)	77 (24.5%)	290 (30.8%)
Insurance status				
Uninsured or Well-Woman	250 (79.1%)	262 (83.2%)	253 (80.6%)	765 (81.0%)
Insured	66 (20.9%)	53 (16.8%)	61 (19.4%)	180 (19.0%)
Primary language at consent				
English	22 (7.0%)	23 (7.3%)	24 (7.6%)	69 (7.3%)
Spanish	294 (93.0%)	292 (92.7%)	291 (92.4%)	877 (92.7%)
1^st^ Degree family history of BC				
No	289 (91.5%)	289 (91.7%)	296 (94.3%)	874 (92.5%)
Yes	27 (8.5%)	26 (8.3%)	18 (5.7%)	71 (7.5%)
Body mass index				
<25	51 (16.2%)	52 (16.5%)	35 (11.1%)	138 (14.6%)
25 to 29	116 (36.8%)	98 (31.1%)	117 (37.3%)	331 (35.1%)
30+	148 (47.0%)	165 (52.4%)	162 (51.6%)	475 (50.3%)
Number of prior mammograms				
0	54 (17.1%)	45 (14.3%)	68 (21.6%)	167 (17.7%)
1	52 (16.5%)	58 (18.5%)	45 (14.3%)	155 (16.4%)
2–4	114 (36.2%)	130 (41.4%)	115 (36.5%)	359 (38.0%)
5+	95 (30.2%)	81 (25.8%)	87 (27.6%)	263 (27.9%)
Ever called back				
Never called back	234 (62.6%)	227 (61.9%)	207 (56.4%)	668 (60.3%)
Called back	73 (19.5%)	82 (22.3%)	81 (22.1%)	236 (21.3%)
Ever had breast biopsy				
No	292 (92.4%)	287 (91.4%)	294 (93.3%)	873 (92.4%)
Yes	24 (7.6%)	27 (8.6%)	21 (6.7%)	72 (7.6%)
Perceived worry about BC				
Rarely or less	186 (59.2%)	194 (61.8%)	160 (51.0%)	540 (57.3%)
At least sometimes	128 (40.8%)	120 (38.2%)	154 (49.0%)	402 (42.7%)
Perceived lifetime risk of BC				
0–10%	229 (75.8%)	233 (77.4%)	231 (75.7%)	693 (76.3%)
>10%	73 (24.2%)	68 (22.6%)	74 (24.3%)	215 (23.7%)
Perceived risk of developing BC				
Not high	302 (97.1%)	300 (96.5%)	295 (95.2%)	897 (96.2%)
At least moderately high	9 (2.9%)	11 (3.5%)	15 (4.8%)	35 (3.8%)
Confidence to have annual mammogram				
Less confident (<8)	31 (9.8%)	22 (7.0%)	30 (9.6%)	83 (8.8%)
Confident (8+)	285 (90.2%)	292 (93.0%)	283 (90.4%)	860 (91.2%)
Likelihood of getting an annual mammogram				
Not at all to somewhat likely	62 (19.7%)	56 (17.9%)	71 (22.7%)	189 (20.1%)
Extremely likely	253 (80.3%)	257 (82.1%)	242 (77.3%)	752 (79.9%)
BI-RAD breast density category at baseline				
Not dense (A/B)	141 (44.6%)	148 (47.0%)	151 (47.9%)	440 (46.5%)
Dense (C/D)	175 (55.4%)	167 (53.0%)	164 (52.1%)	506 (53.5%)

Frequencies not adding up to the column indicate missing data (excluded from the denominator).

BC, breast cancer; LLEAD, Latinas Learning about Breast Density.

As shown in Table 2, the 1-year adherence rate for our cohort was 24.8% (95% confidence interval [95% CI] 22.1%, 27.6%), increasing to 51.7% by year 2 (95% CI 48.1%, 55.4%) (Table 2); however, time to subsequent screening mammogram was not found to differ significantly by study group or among those told they had dense breasts (Supplementary Table S1). Among the 482 Latinas with no subsequent mammogram 10–26 months post-baseline, 215 provided the main reason for not receiving a mammogram in the T2 survey with the majority stating that they forgot, followed by cost issues and waiting to be told by the provider or clinic that it was time for a mammogram. Several women also stated that they were told that they were not due for a mammogram with some saying they needed a mammogram every 3 years.

**Table 2.** Adherence at 1- and 2-Years Post-Baseline Mammogram Overall, by Study Group, and by Baseline MBD Result

Variable	*N*	# Adherent	1-year^a^ % (95% CI)	2-year^b^ % (95% CI)	Hazard ratio (95% CI)	*p*-value
Overall	946	464	24.8% (22.1%, 27.6%)	51.7% (48.1%, 55.4%)		
Study group						0.76
UC	316	151	22.8% (18.2%, 27.4%)	50.5% (44.2%, 56.8%)	Reference	
ENT	315	153	24.1% (19.4%, 28.9%)	51.6% (45.2%, 57.9%)	1.03 (0.82, 1.29)	
INT	315	160	27.6% (22.7%, 32.6%)	53.0% (46.8%, 59.2%)	1.09 (0.87, 1.36)	
MBD						0.65
Not dense	440	224	24.8% (20.7%, 28.8%)	52.3% (47.0%, 57.5%)	Reference	
Dense	506	240	24.9% (21.1%, 28.7%)	51.2% (46.2%, 56.2%)	0.96 (0.80, 1.15)	

^a^ Kaplan–Meier estimate of women receiving a mammogram between 10 and 14 months.

^b^ Kaplan–Meier estimate of women receiving a mammogram between 10 and 26 months.

CI, confidence interval; ENT, enhanced; INT, interpersonal, MBD; mammographic breast density; UC, usual care.

We explored factors associated with adherence to subsequent mammogram for the cohort controlling for age and study group (Table 3). Number of prior mammograms (hazard ratio [HR] = 1.52 [95% CI 1.05 to 2.21], 2.07 [95% CI 1.50 to 2.91], and 3.12 [95% CI 2.18 to 4.52] for 1, 2–4, and 5+ prior mammograms versus 0, respectively, *p* < 0.01), being “extremely likely” to get an annual mammogram (HR 1.43 [95% CI 1.13 to 1.86] *p* < 0.01), and having more confidence (score >=8) to continue mammograms annually (HR 1.47 [95% CI 1.03 to 2.18] *p* = 0.03) were significantly associated with adherence to subsequent mammography. Since intention or likelihood is a predictor of actual behavior,^55^ we explored differences in patient characteristics by future screening intention (Supplementary Table S2). Latinas being “extremely likely” to continue getting an annual mammogram differ from those who reported otherwise in that they are more likely to be older (*p* = 0.02), have had a mammogram prior to study entry (*p* < 0.01), and have more confidence to get an annual mammogram (*p* < 0.01).

**Table 3.** Factor Associations with 1- and 2-Year Adherence Rates, Adjusting for Age and Study Group

Variable	*N*	# Adherent	1-year^a^ % (95% CI)	2-year^b^ % (95% CI)	Hazard ratio^c^ (95% CI)	*p*-value
Education						0.22
Less than high school	651	315	25.2% (21.9%, 28.5%)	51.0% (46.7%, 55.4%)	Reference	
High school or more	290	147	23.8% (18.9%, 28.7%)	53.4% (46.8%, 59.9%)	1.13 (0.93, 1.38)	
Insurance status						1.0
Uninsured or Well-Woman	765	372	24.6% (21.5%, 27.6%)	50.5% (46.5%, 54.5%)	Reference	
Insured	180	92	26.1% (19.7%, 32.5%)	57.8% (48.8%, 66.7%)	1.00 (0.78, 1.27)	
Language of consent form						0.09
English	69	28	15.9% (7.3%, 24.6%)	48.3% (33.5%, 63.2%)	Reference	
Spanish	877	436	25.5% (22.7%, 28.4%)	52.0% (48.3%, 55.8%)	1.38 (0.96, 2.08)	
Body mass index						0.36
<25	138	64	25.4% (18.1%, 32.6%)	47.2% (38.2%, 56.2%)	Reference	
25 to 29	331	170	27.8% (23.0%, 32.6%)	55.7% (49.6%, 61.8%)	1.05 (0.79, 1.41)	
>=30	475	230	22.7% (19.0%, 26.5%)	50.2% (45.1%, 55.3%)	0.91 (0.69, 1.21)	
First-degree family history of BC						0.45
No	874	425	24.5% (21.6%, 27.3%)	51.1% (47.4%, 54.9%)	Reference	
Yes	71	38	28.2% (17.7%, 38.6%)	59.0% (45.4%, 72.5%)	1.14 (0.80, 1.57)	
Number of prior mammograms						<0.001
0	167	51	13.2% (8.0%, 18.3%)	28.8% (21.4%, 36.2%)	Reference	
1	155	65	18.1% (12.0%, 24.1%)	42.6% (34.1%, 51.0%)	1.52 (1.05, 2.21)	
2–4	359	180	22.8% (18.5%, 27.2%)	56.1% (49.8%, 62.3%)	2.07 (1.50, 2.91)	
5+	263	167	38.8% (32.9%, 44.7%)	67.7% (61.0%, 74.4%)	3.12 (2.18, 4.52)	
Ever called back for additional tests						0.38
Never called back	569	299	25.3% (21.7%, 28.9%)	56.9% (52.2%, 61.6%)	Reference	
Called back	204	111	33.3% (26.9%, 39.8%)	57.6% (49.7%, 65.5%)	1.10 (0.88, 1.37)	
Ever had breast biopsy						0.11
No	873	421	23.7% (20.9%, 26.5%)	51.1% (47.3%, 54.9%)	Reference	
Yes	72	43	38.9% (27.6%, 50.1%)	59.7% (47.3%, 72.0%)	1.31 (0.94, 1.78)	
Perceived worry about BC						0.48
Rarely or less	540	263	23.1% (19.6%, 26.7%)	51.6% (46.8%, 56.4%)	Reference	
At least sometimes	402	200	27.1% (22.8%, 31.5%)	52.0% (46.4%, 57.6%)	1.07 (0.89, 1.28)	
Perceived percent lifetime risk of BC						0.60
0–10%	693	345	25.4% (22.2%, 28.6%)	52.8% (48.6%, 57.0%)	Reference	
>10%	215	106	23.7% (18.0%, 29.4%)	50.7% (43.0%, 58.4%)	1.06 (0.85, 1.32)	
Perceived risk of developing BC						0.60
Not high	897	439	24.5% (21.7%, 27.3%)	51.9% (48.1%, 55.6%)	Reference	
At least moderately high	35	19	25.7% (11.2%, 40.2%)	48.9% (32.2%, 65.5%)	1.13 (0.69, 1.75)	
Likelihood of annual mammogram						0.004
Not at all to somewhat likely	189	71	21.2% (15.3%, 27.0%)	38.7% (31.0%, 46.4%)	Reference	
Extremely likely	752	391	25.7% (22.5%, 28.8%)	54.9% (50.8%, 59.0%)	1.43 (1.11, 1.86)	
Confidence to have annual mammogram						0.03
Less confident (<8)	83	30	20.5% (11.8%, 29.2%)	43.4% (29.8%, 57.0%)	Reference	
Confident (8+)	860	432	25.2% (22.3%, 28.1%)	52.5% (48.7%, 56.3%)	1.47 (1.03, 2.18)	
BI-RAD breast density category at baseline						0.44
Not dense (A/B)	440	224	24.8% (20.7%, 28.8%)	52.3% (47.0%, 57.5%)	Reference	
Dense (C/D)	506	240	24.9% (21.1%, 28.7%)	51.2% (46.2%, 56.2%)	1.08 (0.89, 1.30)	

^a^ Kaplan–Meier estimate of women receiving a mammogram between 10 and 14 months.

^b^ Kaplan–Meier estimate of women receiving a mammogram between 10 and 26 months.

^c^ Results of Cox modeling adjusted for age and study group.

BC, breast cancer; CI, confidence interval.

Examining factors associated with adherence stratified by age (Table 4), we found that those receiving more prior mammograms were more adherent compared to those without a prior mammogram for both age groups. No additional factors were significantly associated with adherence among Latinas 50+, while being “extremely likely” to continue getting an annual mammogram (HR 1.61 [95% CI 1.27 to 2.27] *p* < 0.01) and having more confidence to get an annual mammogram (HR 1.76 [95% CI 1.20 to 3.04] *p* = 0.02) were associated with adherence among Latinas >50.

**Table 4.** Factor Associations with 1- and 2-Year Adherence Rates Stratified by Age, Adjusting for Age and Study Group

Age group	Variable	*N*	# Adherent	1-year^a^ % (95% CI)	2-year^b^ % (95% CI)	Hazard ratio^c^ (95% CI)	*p*-value
	Education						
<50	Less than high school	409	182	20.8% (16.9%, 24.7%)	46.5% (41.1%, 51.9%)	Reference	0.58
	High school or more	215	99	21.4% (15.9%, 26.9%)	47.4% (39.9%, 54.8%)	1.07 (0.84, 1.37)	
50+	Less than high school	242	133	32.6% (26.7%, 38.6%)	59.4% (51.9%, 66.8%)	Reference	0.18
	High school or more	75	48	30.7% (20.2%, 41.1%)	72.4% (59.1%, 85.7%)	1.26 (0.90, 1.75)	
	Insurance status						
<50	Uninsured or well-woman	552	247	20.7% (17.3%, 24.0%)	46.6% (42.0%, 51.3%)	Reference	0.67
	Insured	73	35	24.7% (14.8%, 34.5%)	49.8% (36.1%, 63.5%)	1.08 (0.75, 1.52)	
50+	Uninsured or well-woman	213	125	34.7% (28.3%, 41.1%)	61.3% (53.5%, 69.0%)	Reference	0.67
	Insured	107	57	27.1% (18.7%, 35.5%)	64.5% (53.0%, 76.0%)	0.93 (0.67, 1.28)	
	Language at consent						
<50	English	35	13	17.1% (4.7%, 29.6%)	40.4% (21.1%, 59.8%)	Reference	0.38
	Spanish	590	269	21.4% (18.0%, 24.7%)	47.3% (42.8%, 51.8%)	1.27 (0.76, 2.34)	
50+	English	34	15	14.7% (2.8%, 26.6%)	59.4% (35.7%, 83.1%)	Reference	0.14
	Spanish	287	167	34.1% (28.7%, 39.6%)	62.5% (55.7%, 69.2%)	1.47 (0.89, 2.60)	
	Body mass index						
<50	<25	95	43	25.3% (16.5%, 34.0%)	44.3% (33.8%, 54.9%)	Reference	0.89
	25 to 29	217	96	22.6% (17.0%, 28.1%)	48.1% (40.6%, 55.7%)	0.94 (0.66, 1.37)	
	>=30	311	143	19.0% (14.6%, 23.3%)	47.1% (40.9%, 53.3%)	0.92 (0.66, 1.31)	
50+	<25	43	21	25.6% (12.5%, 38.6%)	53.1% (36.7%, 69.5%)	Reference	0.15
	25 to 29	114	74	37.7% (28.8%, 46.6%)	70.7% (60.7%, 80.7%)	1.24 (0.77, 2.06)	
	>=30	164	87	29.9% (22.9%, 36.9%)	56.3% (47.3%, 65.3%)	0.90 (0.57, 1.50)	
	First-degree family history of BC						
<50	No	586	263	21.2% (17.9%, 24.5%)	46.7% (42.2%, 51.3%)	Reference	0.86
	Yes	39	19	20.5% (7.8%, 33.2%)	49.6% (31.9%, 67.3%)	1.04 (0.63, 1.62)	
50+	No	288	162	31.3% (25.9%, 36.6%)	60.9% (54.1%, 67.7%)	Reference	0.36
	Yes	32	19	37.5% (20.7%, 54.3%)	74.0% (52.7%, 95.4%)	1.26 (0.76, 1.97)	
	Number of prior mammograms						
<50	0	158	50	13.9% (8.5%, 19.3%)	29.6% (21.9%, 37.2%)	Reference	<0.001
	1	132	57	21.2% (14.2%, 28.2%)	44.5% (35.4%, 53.7%)	1.52 (1.04, 2.23)	
	2–4	250	121	23.6% (18.3%, 28.9%)	54.2% (46.8%, 61.7%)	1.80 (1.28, 2.57)	
	5+	83	53	26.5% (17.0%, 36.0%)	62.5% (51.3%, 73.6%)	2.49 (1.61, 3.84)	
50+	0–1^d^	32	9	0.0% (NA^e^)	26.2% (9.1%, 43.2%)	Reference	<0.001
	2–4	109	59	21.1% (13.4%, 28.8%)	61.0% (49.3%, 72.7%)	3.23 (1.67, 7.03)	
	5+	180	114	44.4% (37.2%, 51.7%)	69.9% (61.2%, 78.5%)	4.87 (2.57, 10.47)	
	Ever called back for additional tests						
<50	Never called back	362	181	22.7% (18.3%, 27.0%)	53.7% (47.9%, 59.6%)	Reference	0.90
	Called back	102	49	26.5% (17.9%, 35.0%)	48.9% (37.7%, 60.1%)	0.98 (0.71, 1.34)	
50+	Never called back	207	118	30.0% (23.7%, 36.2%)	62.9% (54.7%, 71.2%)	Reference	0.15
	Called back	102	62	40.2% (30.7%, 49.7%)	66.5% (55.9%, 77.1%)	1.26 (0.92, 1.70)	
	Ever had breast biopsy						
<50	No	590	262	20.3% (17.1%, 23.6%)	46.6% (42.1%, 51.1%)	Reference	0.20
	Yes	34	20	35.3% (19.2%, 51.4%)	54.3% (37.1%, 71.4%)	1.38 (0.84, 2.13)	
50+	No	283	159	30.7% (25.4%, 36.1%)	61.4% (54.5%, 68.4%)	Reference	0.34
	Yes	38	23	42.1% (26.4%, 57.8%)	66.9% (48.1%, 85.7%)	1.24 (0.78, 1.89)	
	Perceived worry about BC						
<50	Rarely or less	349	154	18.1% (14.0%, 22.1%)	46.6% (40.7%, 52.5%)	Reference	0.48
	At least sometimes	272	127	25.0% (19.9%, 30.1%)	47.4% (40.8%, 54.0%)	1.09 (0.86, 1.38)	
50+	Rarely or less	191	109	32.5% (25.8%, 39.1%)	61.3% (53.1%, 69.4%)	Reference	0.85
	At least sometimes	130	73	31.5% (23.6%, 39.5%)	63.5% (52.5%, 74.5%)	1.03 (0.76, 1.38)	
	Perceived percent lifetime risk of BC						
<50	0–10%	438	200	21.5% (17.6%, 25.3%)	48.0% (42.8%, 53.2%)	Reference	0.96
	>10%	158	72	19.6% (13.4%, 25.8%)	45.5% (36.7%, 54.3%)	1.01 (0.76, 1.31)	
50+	0–10%	255	145	32.2% (26.4%, 37.9%)	61.6% (54.4%, 68.8%)	Reference	0.40
	>10%	57	34	35.1% (22.7%, 47.5%)	69.3% (53.1%, 85.6%)	1.18 (0.80, 1.69)	
	Perceived risk of developing BC						
<50	Not high	590	264	20.5% (17.3%, 23.8%)	46.9% (42.4%, 51.4%)	Reference	1.0
	At least moderately high	26	13	23.1% (6.9%, 39.3%)	42.3% (23.3%, 61.3%)	1.00 (0.54, 1.68)	
50+	Not high	307	175	32.2% (27.0%, 37.5%)	62.4% (55.8%, 69.0%)	Reference	0.28
	At least moderately high	9	6	33.3% (2.5%, 64.1%)	100.0% (NA^e^)	1.62 (0.63, 3.39)	
	Likelihood of annual mammogram						
<50	Not at all to somewhat likely	132	42	16.7% (10.3%, 23.0%)	32.1% (23.6%, 40.7%)	Reference	0.003
	Extremely likely	489	239	22.3% (18.6%, 26.0%)	50.8% (45.8%, 55.7%)	1.61 (1.17, 2.27)	
50+	Not at all to somewhat likely	57	29	31.6% (19.5%, 43.6%)	54.3% (38.6%, 70.1%)	Reference	0.45
	Extremely likely	263	152	31.9% (26.3%, 37.6%)	63.1% (56.1%, 70.2%)	1.16 (0.79, 1.77)	
	Confidence to have annual mammogram (scale 0 to 10)						
<50	Less confident (<8)	56	16	12.5% (3.8%, 21.2%)	33.4% (17.9%, 49.0%)	Reference	0.02
	Confident (8+)	566	264	21.9% (18.5%, 25.3%)	48.1% (43.6%, 52.7%)	1.76 (1.10, 3.04)	
50+	Less confident (<8)	27	14	37.0% (18.8%, 55.3%)	64.4% (41.0%, 87.8%)	Reference	0.67
	Confident (8+)	294	168	31.6% (26.3%, 36.9%)	61.7% (55.0%, 68.4%)	1.13 (0.68, 2.04)	
	BI-RAD breast density category at baseline						
<50	Not dense (A/B)	238	107	17.2% (12.4%, 22.0%)	44.8% (37.8%, 51.8%)	Reference	0.36
	Dense (C/D)	387	175	23.5% (19.3%, 27.7%)	48.1% (42.5%, 53.7%)	1.12 (0.88, 1.43)	
50+	Not dense (A/B)	202	117	33.7% (27.1%, 40.2%)	61.7% (53.7%, 69.6%)	Reference	0.97
	Dense (C/D)	119	65	29.4% (21.2%, 37.6%)	62.6% (51.5%, 73.8%)	1.01 (0.74, 1.36)	

^a^ Kaplan–Meier estimate of women receiving a mammogram between 10 and 14 months.

^b^ Kaplan–Meier estimate of women receiving a mammogram between 10 and 26 months.

^c^ Results of Cox modeling adjusted for age (dichotomously within each age subset) and study group.

^d^ Combining 0–1 prior mammograms together in the older age group due to sparse data (in those with zero prior mammograms, there were only nine participants with 1F/U mammogram).

^e^ Confidence interval not calculated due to lack of variability.

BC, breast cancer; CI, confidence interval.

## Discussion

This is one of the first studies to examine the effectiveness of three BD educational interventions on underserved Latina’s adherence to subsequent mammography. Overall, approximately half (51.7%) of the Latinas enrolled in our study were adherent to their subsequent mammogram within 2 years with no significant differences observed by study group, regardless of MBD status. After adjusting for age and study group, number of prior mammograms, likelihood of getting an annual mammogram (intention), and confidence to get an annual mammogram (self-efficacy) reported at baseline were significant drivers of adherence. We did not detect significant differences in variables previously reported to contribute to adherence, like insurance status, though open-ended responses from nonadherent Latinas self-reported cost being one of the main reasons for not getting a mammogram.

Educational approaches deployed in this trial, including Promotora-led one-on-one education, have shown to significantly improve proximal outcomes of BD notification including knowledge and awareness of BD;^38,51^ however, we did not observe significant differences in adherence to subsequent mammography across our three educational approaches regardless of MBD status. Though we observed a magnitude of increase between 2% and 4% for our Promotora-led educational group compared to our enhanced and usual care approaches, this is arguably not of great clinical significance. These findings may be due to limited power to detect small differences across study arms, but almost half (46.5%) of Latinas enrolled in our study did not have dense breasts at baseline. While our educational materials included information on additional risk factors for BC and motivational messaging around the importance of getting a mammogram, the primary focus was to educate about BD. It is possible Latinas without dense breasts perceived their risk of BC to be less and may be less motivated to get a mammogram,^18,56^ but we did not observe any significant differences in subsequent mammography by study group even among a subset of Latinas with dense breasts. These findings emphasize the limitations of educational interventions on behavioral outcomes among populations susceptible to disparities in BC outcomes.

Over 80% of Latinas enrolled in our study had at least one prior mammogram, but only 24.8% received a subsequent mammogram at 1 year increasing to 51.7% at 2-years post-enrollment. Considering that two-thirds of women enrolled in the study were under the age of 50, these findings may reflect variation in screening initiation and frequency across guideline recommendations.^44,45,57,58^ Yet, our study site recommended annual mammography for all patients regardless of MBD status. Similar to prior studies, we found that self-reported cost barriers,^59–61^ forgetting to schedule an appointment,^61^ and lack of a provider recommendation^59,62^ were main reasons for Latinas not getting their annual mammogram. Multilevel interventions that include Promotora-led education (beyond a single risk factor) combined with strategies shown to be effective in increasing mammography use, such as patient reminders,^62^ provider recommendations,^63^ and addressing barriers to care are likely needed to facilitate mammography adherence in underserved Latinas.

Our findings support existing literature that having more prior mammograms^59,62,64–66^ and higher levels of future screening intent and self-efficacy^65,67^ may be significant drivers of adherence to subsequent mammography. Prior studies show that Latinas with a prior or recent mammogram were more likely to hold stronger intentions to obtain a mammogram in the future.^59,64^ Moreover, as women age, they have more opportunities to participate in and overcome barriers to mammography screening,^67^ making screening a routine part of care.^68–70^ For younger Latinas, mammography screening may be a relatively new behavior that is often not perceived as a priority.^71^ With guideline recommending mammography screening starting by age 40,^72^ efforts to improve adherence to subsequent mammography should consider targeting relatively new screeners to help improve screening intention and self-efficacy to get a mammogram.

The strengths of this study include the randomized trial design and—ascertaining adherence using both EHR and self-reported survey data to account for Latinas receiving a mammogram within and outside our setting. However, all women were recruited from the same FQHC with similar sociodemographic characteristics (e.g., Latina, Spanish speaking), limiting generalizability. Additionally, the majority of our sample was under the age of 50 and nearly half without dense breasts. While this provided a unique opportunity to explore the effects of notification for all women regardless of age or MBD status and is consistent with legislation enacted in neighboring states, it is possible that the enhanced and interpersonal intervention arms lacked relevance reducing effects. However, subgroup analyses examining the effect of our intervention arms by MBD status did not show significant effects. It is also possible that our inability to detect associations may be due to reduced sample size to account for the effect of COVID-19 on mammography screening. While the need for additional screening options was discussed for those with MBD, we were unable to ascertain adherence to supplemental screening since supplemental screening was not available on site. Finally, these data are limited to intervention effects on adherence pre-pandemic and may not be representative of the changing BD policy landscape or the role of COVID-19 on screening behavior more broadly. Despite these limitations, this is one of the first randomized controlled trials showing the effect of BD educational approaches on both behavioral outcomes among Latinas receiving care at a FQHC.

The FDA estimated that BD notification would lead to a reduction in mortality and BC treatment costs, but these findings highlight the complexity and limitations of BD notification for Spanish-speaking Latinas receiving care from FQHCs. In summary, our findings suggest that BD educational interventions alone are unlikely to achieve the desired behavioral effects, including future repeat mammography screening. Multilevel interventions that combine educational approaches with patient reminders, provider recommendations, and support to overcome barriers to care, including access to and cost burden associated with mammography and supplemental imaging, are likely needed to supplement BD notifications, particularly for underserved, Latinas. Moreover, future efforts should also consider targeting younger women new to mammography screening or without a recent mammogram to increase behavioral intention and self-efficacy to undergo annual screening.

## Data Availability

The data underlying this article cannot be publicly shared due to existing consent and Institutional Review Board constraints. Additional summary-level data without individual data may be shared upon request and permission from community partners who supported this project.
