# Supplementary material for: Effectiveness of Breast Density Educational Interventions on Mammography Screening Adherence Among Underserved Latinas: A Randomized Controlled Trial
Source: J Womens Health (Larchmt). 2025 Apr 2;34(4):491–503. doi: 10.1089/jwh.2024.0273 (PMC12408887; doi:10.1089/jwh.2024.0273)
Supplement: Supplementary Table S1 [file jwh.2024.0273_supplementarytables1.docx]

| **Supplement Table 1.** Adherence at 1- and 2-years post baseline mammogram among women with dense breasts by study group | | | | | | | |
| --- | --- | --- | --- | --- | --- | --- | --- |
| **Baseline MBD** | **Study Group** | **N** | **# Adherent** | **1-year^a^ % (95% CI)** | **2-year^b^ % (95% CI)** | **Hazard Ratio**  **95% CI** | **P-value** |
| Dense | Usual | 175 | 77 | 23.4% (17.2%, 29.7%) | 47.4% (39.2%, 55.7%) | Reference | 0.62 |
|  | Enhanced | 167 | 78 | 24.0% (17.5%, 30.4%) | 52.9% (43.9%, 61.9%) | 1.05 (0.77, 1.44) |  |
|  | Interpersonal | 164 | 85 | 27.4% (20.6%, 34.3%) | 53.3% (44.7%, 61.8%) | 1.16 (0.85, 1.58) |  |
|  |  |  |  |  |  |  |  |
| Not Dense | Usual | 141 | 74 | 22.0% (15.1%, 28.8%) | 53.5% (44.1%, 62.9%) | Reference | 1.0 |
|  | Enhanced | 148 | 75 | 24.3% (17.4%, 31.2%) | 50.3% (41.3%, 59.2%) | 1.01 (0.73, 1.39) |  |
|  | Interpersonal | 151 | 75 | 27.8% (20.7%, 35.0%) | 52.7% (43.7%, 61.7%) | 1.01 (0.73, 1.39) |  |
| Abbreviations: CI, confidence interval  ^a^Kaplan-Meier estimate of women receiving a mammogram between 10 and 14 months (Kaplan-Meier estimate)  ^b^Kaplan-Meier estimate of women receiving a mammogram between 10 and 26 months (Kaplan-Meier estimate) | | | | | | | |
