# Supplementary material for: Effectiveness of Breast Density Educational Interventions on Mammography Screening Adherence Among Underserved Latinas: A Randomized Controlled Trial
Source: J Womens Health (Larchmt). 2025 Apr 2;34(4):491–503. doi: 10.1089/jwh.2024.0273 (PMC12408887; doi:10.1089/jwh.2024.0273)
Supplement: Supplementary Table S2 [file jwh.2024.0273_supplementarytables2.docx]

| **Supplement Table 2.** Differences in future screening intention by LLEAD participant characteristics | | | |
| --- | --- | --- | --- |
|  | Not at all to somewhat likely (N=189) | Extremely likely (N=752) | P-value |
| **Age** |  |  | 0.02 |
| 40-44 | 86 (45.5%) | 253 (33.6%) |  |
| 45-49 | 46 (24.3%) | 236 (31.4%) |  |
| 50-54 | 30 (15.9%) | 138 (18.4%) |  |
| 55+ | 27 (14.3%) | 125 (16.6%) |  |
|  |  |  |  |
| **Education** |  |  | 0.06 |
| Less than high school | 141 (75.0%) | 507 (67.8%) |  |
| High school or more | 47 (25.0%) | 241 (32.2%) |  |
|  |  |  |  |
| **Body Mass Index** |  |  | 0.29 |
| <25 | 26 (13.8%) | 112 (14.9%) |  |
| 25 to <30 | 75 (39.9%) | 254 (33.8%) |  |
| >=30 | 87 (46.3%) | 385 (51.3%) |  |
|  |  |  |  |
| **Insurance Status** |  |  | 0.10 |
| Uninsured or Well-Woman | 160 (85.1%) | 600 (79.8%) |  |
| Insured | 28 (14.9%) | 152 (20.2%) |  |
|  |  |  |  |
| **1^st^ Degree Family History of BC** |  |  | 0.19 |
| No | 179 (94.7%) | 690 (91.9%) |  |
| Yes | 10 (5.3%) | 61 (8.1%) |  |
|  |  |  |  |
| **Primary language at consent** |  |  | 0.40 |
| English | 11 (5.8%) | 57 (7.6%) |  |
| Spanish | 178 (94.2%) | 695 (92.4%) |  |
|  |  |  |  |
| **Number of prior mammograms** |  |  | <0.001 |
| 0 | 54 (28.7%) | 112 (14.9%) |  |
| 1 | 33 (17.6%) | 120 (16.0%) |  |
| 2-4 | 67 (35.6%) | 291 (38.7%) |  |
| 5+ | 34 (18.1%) | 228 (30.4%) |  |
|  |  |  |  |
| **Ever had breast biopsy** |  |  | 0.18 |
| No | 178 (94.7%) | 690 (91.8%) |  |
| Yes | 10 (5.3%) | 62 (8.2%) |  |
|  |  |  |  |
| **Perceived worry about BC** |  |  | 0.35 |
| Rarely or less | 101 (54.3%) | 436 (58.1%) |  |
| At least sometimes | 85 (45.7%) | 315 (41.9%) |  |
|  |  |  |  |
| **Perceived lifetime risk of BC** |  |  | 0.75 |
| 0-10% | 138 (75.4%) | 551 (76.5%) |  |
| >10% | 45 (24.6%) | 169 (23.5%) |  |
|  |  |  |  |
| **Perceived risk of developing BC** |  |  | 0.69 |
| Not high | 177 (96.7%) | 715 (96.1%) |  |
| At least moderately high | 6 (3.3%) | 29 (3.9%) |  |
|  |  |  |  |
| **Confidence to have annual mammogram (scale 0-10)** |  |  | <0.001 |
| Less confident (<8) | 40 (21.2%) | 43 (5.7%) |  |
| More confident (8+) | 149 (78.8%) | 706 (94.3%) |  |
|  |  |  |  |
| Abbreviations: BC, breast cancer |  |  |  |
